# Feeding Enrichment in a Captive Pack of European Wolves (*Canis Lupus Lupus*): Assessing the Effects on Welfare and on a Zoo’s Recreational, Educational and Conservational Role

**DOI:** 10.3390/ani9060331

**Published:** 2019-06-08

**Authors:** Giacomo Riggio, Chiara Mariti, Chiara Boncompagni, Simone Corosaniti, Massimiliano Di Giovanni, Asahi Ogi, Angelo Gazzano, Robert Thomas

**Affiliations:** 1 Vethos, Via E. Besta 46, 00167 Rome, Italy; giacomoriggio@gmail.com (G.R.); boncompagnichiara@gmail.com (C.B.); scorosaniti@hotmail.it (S.C.); 2 Department of Veterinary Sciences, University of Pisa, Viale delle Piagge 2, 56124 Pisa, Italy; chiara.mariti@unipi.it (C.M.); asahi.ogi@vet.unipi.it (A.O.); 3 Research Department, Fondazione Bioparco di Roma, Viale del Giardino Zoologico 20, 00197 Rome, Italy; massimiliano.digiovanni@bioparco.it; 4 Deanery of Biomedical Sciences, University of Edinburgh, Teviot Place, Edinburgh EH8 9AG, UK; rob.thomas@ed.ac.uk

**Keywords:** zoo, wolf behaviour, animal welfare, visitor, conservation, education

## Abstract

**Simple Summary:**

Feeding enrichment is widely used to improve the welfare of zoo animals, but it may also affect zoo visitors’ experience and perception of the animals. The objective of this study was to assess the effects of a naturalistic and a non-naturalistic feeding enrichment program, on both wolf behaviour and visitors’ interest in the exhibit. A questionnaire was administered to visitors with the aim of assessing whether our feeding enrichment programs might affect their perception of captive wolf welfare as well as their attitude towards wolf conservation issues. Our findings suggest that, although wolves seemed to benefit from enrichment, their behavioural responses were highly variable among individuals. Visitors’ interest in the exhibit and perception of captive wolf welfare improved by observing the wolves interacting with food, especially when novel feeding objects were provided. Finally, their attitude towards wolf conservation issues did not change in relation to enrichment, but improved when they observed the wolves performing feeding-related behaviours. These findings may help zoos implement enrichment programs that are effective for enhancing their wolves’ welfare as well as their recreational and educational role.

**Abstract:**

This study investigated the effects of two feeding enrichment programs on the behaviour of a captive pack of European wolves (*Canis lupus lupus*) and their correlation with both zoo visitors’ interest towards the exhibit and their overall perception of the species. Behavioural data (exploration, stereotypies, social interactions, activity/inactivity rates) were collected on four male wolves during four two-week long phases: initial control, hidden food, novel object, final control. Three observation sessions were performed daily: before, during and after feeding. Number of visitors and their permanence in front of the exhibit were recorded. After watching the wolves, visitors were asked to fill out a brief questionnaire in order to investigate their perception of captive wolf welfare, as well as their attitude towards wolf conservation issues. Despite the high inter-individual variability in their behavioural response, all wolves seemed to benefit from feeding enrichment. With regard to visitors, interest in the exhibit increased when enrichment was provided. Visitors’ perception of the level of welfare of wolves improved if they attended a feeding session, especially during the novel object phase. Visitors’ attitude towards wolf conservation issues also improved during feeding sessions, regardless of enrichment provision.

## 1. Introduction

Wolf conservation has always been a very controversial topic because of the impact of wolf predatory activity on livestock [1]. If on one hand, wolves held in zoos may help raise public awareness on the need to preserve the species, on the other they may have their welfare compromised by the impossibility to perform natural behaviours, including predation itself [2,3].

In the Carnivora, predatory motivation influences the individual’s behaviour from the early stages of its psycho-physical development, even in apparently extraneous contexts than those related to food consumption (i.e., play) [4]. Furthermore, in social species like the wolf, group hunting may also have a cohesive function among members of the pack [5,6].

For management and ethical reasons, captive carnivores are rarely given the opportunity to perform predatory behaviour, as food is often served ready to be consumed [7,8,9]. Constantly preventing animals from appeasing their predatory motivation may lead to chronic frustration and stress [10]. Abnormal behaviours may develop as coping strategies in response to the stressful environment [11].

Feeding enrichment programs aim to promote the expression of natural behaviours and the interactions with a more adequately stimulating environment [12,13]. In captive carnivores, such programs are often aimed to stimulate at least some phases of predatory behaviour [14]. The ultimate goal is, of course, that of enhancing the animals’ level of welfare [15,16]. Several behavioural indicators of welfare have been used to assess the effects of feeding enrichment programs. In carnivores, feeding enrichment has been shown to decrease stereotypies [14,17], improve behavioural diversity [18,19], increase exploration [20] and activity levels [21].

Improving animal welfare may be beneficial not just for the animals, but for zoo visitors [22,23]. For instance, animals that interact with their environment have been shown to increase visitor interest in the exhibit [22,24,25,26]. Furthermore, sleeping or pacing animals objectively provide less information about the behavioural repertoire of the species and may fail to meet visitors’ expectations of the animals’ behaviours [27]. Visitors who have a negative experience may leave the zoo with wrong or little, if at all, new knowledge on the species and a sense of frustration for not having their expectations met [8,28]. On the opposite, more active and naturally behaving animals have been shown to improve visitors’ perception of animal welfare and, as a consequence, their perception of the educational importance of zoos [8,29,30].

This study aimed to implement two distinct feeding enrichment procedures in a captive pack of European wolves (*Canis lupus lupus*) in order to (1) simultaneously assess their effects on both captive wolves’ behaviour and visitors’ interest towards the exhibit, (2) assess the possible correlation between enrichment and visitors’ perception of captive wolf welfare, as well as their attitude towards wolf conservation.

## 2. Materials and Methods

### 2.1. Time and Setting

**Figure 1:**
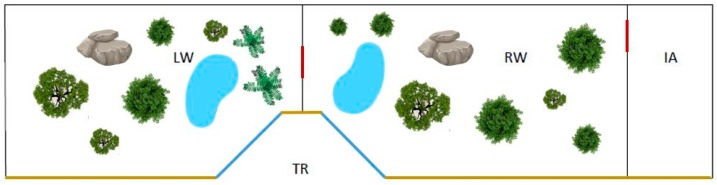
Simplified map of the wolf exhibit. LW = left wing, RW = right wing, IA = isolation area, TR = trapezoidal recess. Red lines = sliding doors, blue lines = windows, yellow lines = wooden palisade.

The study was carried out at the Bioparco of Rome (Italy) for two consecutive months, between March and May 2017. The wolf enclosure is one of the largest exhibits of the park. It is a 200 m^2^ area, surrounded by a 3 m high wooden palisade on one long side (the visitor side). A central 10 m^2^ trapezoidal recess virtually divides the enclosure in a right and a left area. Windows in the two diagonal sides of the recess allow visitors to observe the animals in both areas. When necessary, metal sliding doors are used to physically divide the enclosure in three smaller areas (Figure 1).

### 2.2. Animals

Experimental subjects were four European wolves (*Canis lupus lupus*). All of them were un-neutered male siblings: wolf 1, 2 and 3 were 8 years old at the time of the study and belonged to the same litter, while wolf 4 was 1 year younger. All of them had always lived together and they were moved together from another zoo where they were born and raised. They were fed once a day for 6 days a week and their diet comprised either chunks of buffalo or cow meat, or entire rabbits or quails.

### 2.3. Experimental Design

The experimental protocol was scheduled into four different phases: initial control (ICp), hidden food (HFp), novel object (NOp), final control (FCp). Each phase lasted 2 weeks. ICp, in which no enrichment was administered, served as a baseline control. Food was administered as usual, in the small area at the edge of the right wing of the enclosure. During HFp, food was divided into 12 pieces, regardless of its nature. Four of them were singly buried 10 to 15 cm deep in holes purposely dug by the keepers. Four were suspended 2–3 m-high on tree branches. The remaining pieces were singly inserted deep in the middle of four woodpiles previously built in each wing of the enclosure. These piles had been introduced into the enclosure 2 months before the beginning of the study in order to allow the wolves to habituate to the new elements. During NOp food had to be wrapped in eight canvas bags in order to make it more difficult for the wolves to reach for it. In addition, a hard-plastic feeding ball (Aussiedog^®^) filled with smaller chunks of meat had to be randomly left around the area. Again, during FCp, no enrichment was provided. During this phase, which served as final double control, food was administered using the same procedure as in ICp. Feeding procedures were standardized across the four phases. Keepers would alternately lock the wolves in the left or right area, by using the central metal sliding door. Hence, they would enter the other area in order to clean it and place food. At the end of these procedures they would step out, lock the cage, and free the wolves. Once the sliding door was opened, keepers had 2 min to walk out of sight to the wolves. The study was observational in nature. The animals involved in this study were housed at the zoo and were part of a program of enrichment promoted by the zoo itself, i.e., the procedures were thus not carried out for research purposes. No animal care license nor approval of ethical committees were therefore needed.

### 2.4. Wolf Data Collection

Behavioural observations were carried out daily, 5 days a week, from Tuesday to Saturday. Every day, three observation sessions were performed before, during and after feeding. Each session lasted 1 h, plus a 1-h interval between consecutive sessions, for a total of 3 h per day. In order to meet keepers’ working and break hours and zoo opening hours, but still avoid predictability by wolves on feeding times, observations could start at three different times of the day: 9.30, 11.00, or 12.15. Daily observation starting times were randomly set. Behavioural data were collected through instantaneous scan sampling technique [31]. During each hour of observation, the behaviour of each wolf was recorded every 1 min. The ethogram comprised 16 behavioural categories (Table 1) and was obtained by integrating behaviours described in scientific literature [5,18,32,33,34] with direct behavioural observations over a 7-day period, prior to the study. Since behavioural data from the first observation session on 29 March 2017 had not been recorded, data from the entire day were eliminated.

### 2.5. Visitor Data Collection

Data from visitors were collected by an additional observer using the same observation schedule used for the wolves. Both the number of visitors and the duration of their permanence in front of the exhibit were recorded [35]. The number of visitors in front of the enclosure was recorded by instantaneous scan sampling with 1-min intervals for the entire hour of observation for each session. Children up to 16 years were excluded from the count. The amount of time (seconds) spent in front of the exhibit by every third visitor (excluding children) was also recorded [28,29,36,37]. Visitor permanence recording began when the selected subject walked across the virtual line that delimited the wolf exhibit observation zone (trapezoidal recess) and ended when they crossed it the opposite way.

### 2.6. Questionnaire

When visitors exited the observation zone, a researcher asked them (not children) to fill in a brief questionnaire on wolves. Visitors selected for filling the questionnaire and those selected for recording their permanence in front of the exhibit were not necessarily the same. All visitors were asked to fill the questionnaire although not all of them would accept (respondents *n* = 630). The questionnaire consisted of two sections. The first section investigated on demographic information, which are summarized in Table 2. The second section consisted of 11, 1–5 Likert-scale items partially based on surveys used in previous studies [28,38] (Table 3). Visitors could respond by marking with an X a number from 1 to 5 depending respectively on the degree of disagreement/agreement with the corresponding statement, with 1 meaning “strongly disagree” and 5 meaning “strongly agree.” Items’ positivity and negativity was intended in relation to the quality of visitors’ perception towards wolves.

### 2.7. Statistical Analysis

All the statistical analyses were run with the software SPSS Statistic 17.0 (Chicago, IL, USA).

Due to the small sample size and the possible individual differences in behavioural responses to enrichment, data were analysed separately for each wolf, which acted as its own control. For analysis purposes, all behaviours except stereotypies were grouped into broader macro-categories (Table 1). Exploratory behaviour was also analysed singularly. In order to assess potential differences in each wolf behaviour among phases and among observation sessions, the Kruskal Wallis test and then Wilcoxon signed rank test were applied (*p* < 0.05 multiple comparison corrections were performed using the Benjamini–Hochberg procedure). The Wilcoxon signed rank test was also used to compare data obtained from feeding sessions in the first and second week for both hidden food and novel objects phases, with the aim of assessing a possible effect of the decreased novelty of the enrichment. Potential correlations between number of visitors in front of the exhibit and respectively phase, session and number of active wolves, were analysed using the Spearman test (*p* < 0.05). The Kruskal Wallis test was used to investigate potential differences in visitor stay time in front of the exhibit across observation sessions and experimental phases. When appropriate, pair-wise comparisons were carried out using the Mann–Whitney U test (*p* < 0.05) for independent variables.

As for the questionnaire, descriptive statistics was used for demographic information. Furthermore, all the negative items on the Likert-scale questionnaire were reverse-scaled to match with the score of positive items. Principal Component Analysis (PCA) was used to identify a possible underlying structure across the items; promax rotation with a correlation matrix was used.

Items were included in a component if their loading in that component was >0.50 and their loading in the other components was <0.25. The three principal components found were further analysed by using a Kruskal Wallis Test and then a Mann–Whitney U test (*p* < 0.05) in order to assess differences among phases and sessions.

## 3. Results

### 3.1. Wolf Behavioural Results

Within each enrichment phase, none of the comparisons between the first and second week of observation during feeding session revealed a statistically significant difference. Activity and inactivity rates did not significantly differ across phases, for any of the wolves. Significant results were obtained for stereotypies, social behaviour and exploration, as reported below.

**Figure 2:**
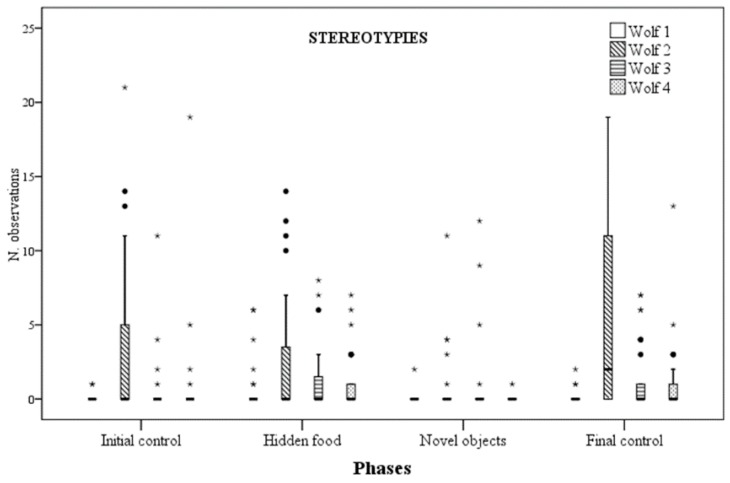
Number of times stereotypic behaviour was observed for each wolf across the four experimental phases.

A significant difference was found in the rate of stereotypic behaviours for wolf 2 and wolf 4 across phases. For wolf 2, stereotypy rates were significantly lower in the NOp when compared with the HFp (*p* = 0.012) and the FCp (*p* = 0.001). Similarly, wolf 4 stereotypy rates were significantly lower during the NOp when compared with the HFp (*p* = 0.003) and the FCp (*p* = 0.001). In addition, wolf 4 stereotypy rates tended to be higher in FCp compared to ICp (*p* = 0.054) (Figure 2).

**Figure 3:**
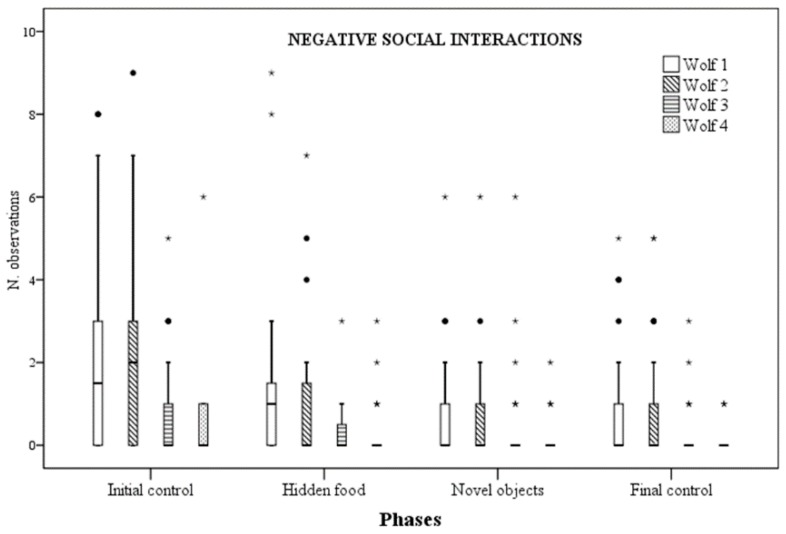
Number of times negative social interactions were observed for each wolf across the four experimental phases.

**Figure 4:**
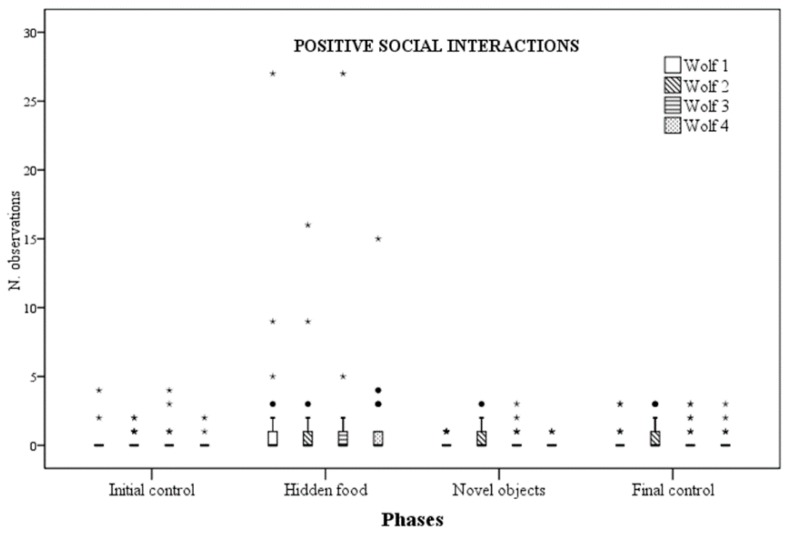
Number of times positive social interactions were observed for each wolf across the four experimental phases.

Social behaviour rates significantly differed for wolf 1, wolf 2 and wolf 4 across phases, whilst no difference was observed for wolf 3. More specifically, for wolf 1, negative social behaviour rates were significantly lower during NOp compared to ICp (*p* = 0.013). However, they were also lower during FCp compared to ICp (*p* = 0.025). Similarly, for wolf 2, negative social behaviour rates were significantly lower during NOp, if compared with ICp (*p* = 0.012). However, they were also different between control phases, being significantly lower during the FCp (*p* = 0.042) (Figure 3). As for wolf 4, statistical differences were instead found for positive social behaviour rates, that resulted higher during HFp than ICp (*p* = 0.011) and NOp (*p* = 0.024) (Figure 4).

**Figure 5:**
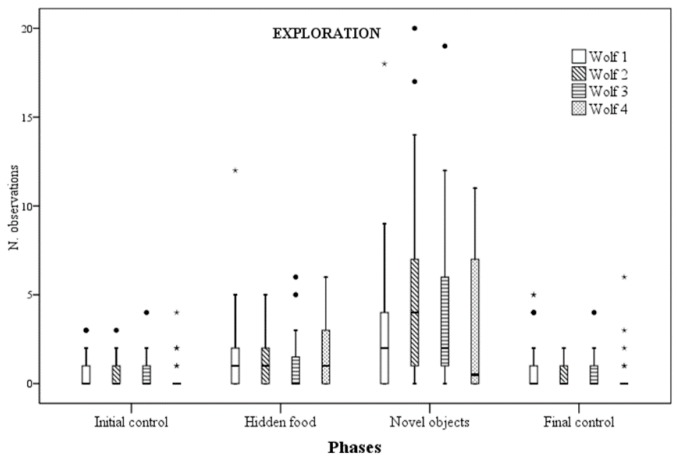
Number of times exploratory behaviour was observed for each wolf across the four experimental phases.

For all wolves there were no statistically significant differences in exploratory behaviour rates between initial and final control phases. Wolf 1 exploratory behaviour rates were significantly higher during the NOp when compared with both ICp (*p* = 0.008) and FCp (*p* = 0.029). For wolf 2, they were significantly higher in NOp when compared with any other phases (NOp versus ICp: *p* = 0.001; NOp versus HFp: *p* = 0.001; NOp versus FCp: *p* = 0.001), as well as in HFp when compared with ICp (*p* = 0.043). Additionally, for wolf 3 exploration was significantly higher in NOp when compared with any other phases (NOp versus ICp: *p* = 0.001; NOp versus HFp: *p* = 0.002; NOp versus FCp: *p* = 0.001). Finally, for wolf 4, exploratory behaviour rates were significantly higher in both the enrichment phases when compared with the control phases (HFp versus ICp: *p* = 0.001; HFp versus FCp: *p* = 0.001; NOp versus ICp: *p* = 0.005; NOp versus FCp: *p* = 0.003) (Figure 5).

### 3.2. Visitors’ Interest Results

**Figure 6:**
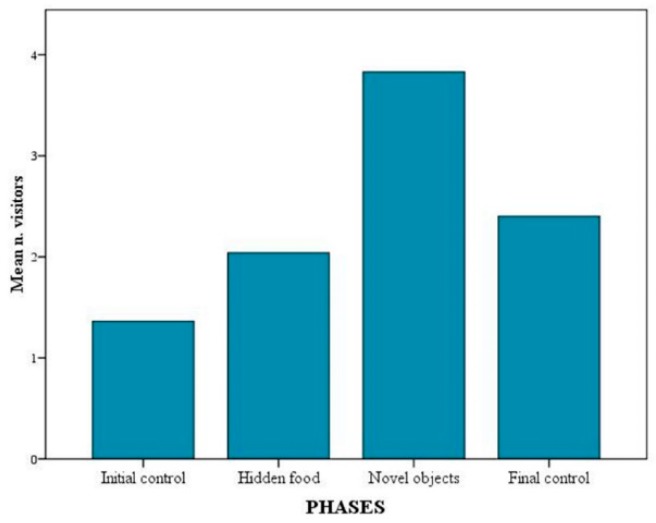
Mean number of visitors in front of the exhibit across the four experimental phases.

**Figure 7:**
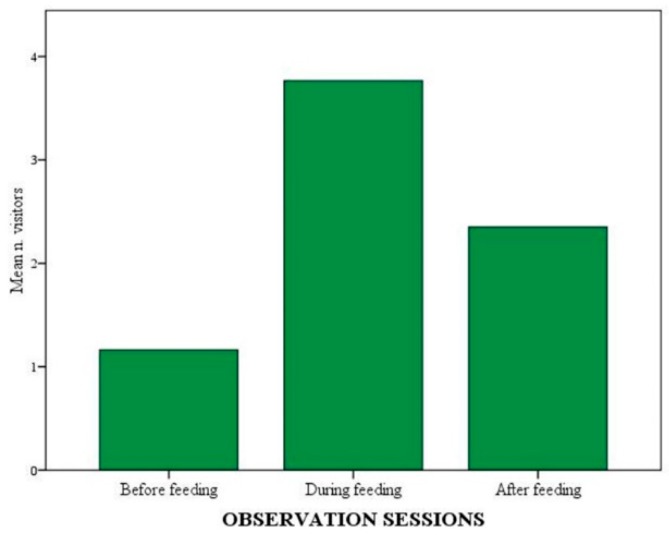
Mean number of visitors in front of the exhibit across the three daily observation sessions.

A positive correlation was found between mean number of visitors in front of the exhibit and experimental phase (Rho value > 0.001), as well as mean number of visitors and observation session (Rho value > 0.001). The mean number of visitors was the highest during NOp (*p* < 0.001). Unexpectedly, it was also higher during FCp phase than HFp (*p* < 0.001) (Figure 6). As for the sessions, the highest mean number of visitors was found during feeding sessions (*p* < 0.001), regardless of the use of enrichment (Figure 7). Moreover, a positive correlation between the number of active wolves and the number of visitors in front of the exhibit was found (*p* < 0.001).

Time spent by visitors in front of the exhibit differed significantly across phases. It was longer during NOp than during any other phase (*p* < 0.001). It was also longer during HFp when compared with both control phases (*p* < 0.001). No significant difference was found between control phases. Furthermore, visitors’ permanence in front of the exhibit also differed across observation sessions, being the longest during feeding sessions (*p* < 0.001) and longer before feeding than after feeding (*p* = 0.016).

### 3.3. Questionnaire Results

The 11, 1–5 Likert-scale items from the questionnaire were analysed using a Principal Component Analysis (PCA) with promax rotation (*p* < 0.05). The component correlation matrix (Table 4) did not show a correlation between the three components. The PCA model was adequate as verified by the Keiser Meyer Olkin (KMO) test (KMO = 0.694; *p* < 0.001). Three principal components (PC1, PC2 and PC3) were identified that explained 49.895% of the variation (Table 5). PC1 was characterized by those items relating to the respondent’s perception of wolf welfare in zoos. PC2 was characterized by those items relating to the respondent’s perception of wolves as a threat to humans and human activities. Finally, PC3 comprised those items relating to respondent’s attitude towards wild wolf population management measures. No significant differences in questionnaire responses were found when data from the three daily sessions were compared across phases. On the contrary, differences were found for PC1 and PC3 across sessions. In particular, respondents scored significantly higher for PC1 and PC3 when questionnaires were administered during feeding when compared with sessions before feeding (PC1: *p* = 0.016; PC3: *p* = 0.010), suggesting that their perception of captive wolves’ welfare and their attitude towards wild population management measures improved by watching the animals interacting with food, regardless of the use of enrichment. However, by comparing questionnaire responses during the sole feeding sessions across the four experimental phases, we found that respondents scored higher for PC1 when they observed the wolves get fed during NOp and HFp rather than during ICp.

## 4. Discussion

### 4.1. Wolves

In this study, none of our significant results regarding wolf behaviour were consistent across all four experimental subjects. This suggests individual factors played a major role in determining the wolves’ responses to feeding enrichment. Three behavioural categories were used to assess wolf responses to enrichment: stereotypies, quality of social interactions and exploration.

Stereotypic behaviours are probably the most used behavioural indicator of welfare in captive animal studies [39,40]. They have been defined as “repetitive, invariant behavioural patterns with no apparent goal” [41]. In this study, two distinct behaviours fit such definition: pacing and jumping against the fence. The latter was performed by only one individual, namely wolf 2. Both of them fall in the category of “locomotor stereotypies,” which are the most common type of stereotypy in captive carnivores [42,43]. In order to meet the criteria of “repetitive, invariant behavioural pattern” we reported such behaviours only when they were performed for at least three consecutive times [27,42]. In this study, two out of four wolves showed a significantly lower rate of stereotypic behaviours when novel artificial feeding objects were provided. Although the underlying processes of stereotypic behaviour in zoo animals are not yet clearly understood, one of the most widely accepted theories is that it represents a copying strategy performed by an individual in response to a chronic stress condition generated by a sub-optimal and restrictive environment [2,39,42,44,45]. Therefore, a reduction in the rate of stereotypic behaviours may be suggestive of increased welfare, especially when other behavioural indicators of welfare improve concurrently [39].

Quality of social interactions was assessed classifying social behaviour in either “positive” or “negative” [32]. Social play and affiliative interactions were considered to be positive, whereas agonistic and aggressive interactions were considered to be negative [32]. Again, in the context of our study, a quite high degree of individual variability in wolves’ behavioural responses to enrichment was observed. While wolf 4 increased positive social interactions when food was hidden around the enclosure, wolf 1 and wolf 2 showed a decrease in negative interactions when artificial feeding objects were provided, whereas wolf 3 showed no significant change in any enrichment programs. As by nature, social interactions occur among more individuals, the interpretation of results needs to take into consideration the possible reciprocal influence of the behaviour of the various parties involved. In this case, the decrease of negative social interactions observed in wolf 1 and 2 are likely to be the result of the decrease of negative social behaviours in one wolf; the parallel trend in the second wolf probably mirrors the first decrease, as a consequence of receiving fewer negative interactions. However, looking at the whole unit, both the direct and indirect impact of enrichment can be regarded as beneficial.

An increase in exploration when novel feeding objects were provided was the only behavioural response common to the four wolves. Nevertheless, for wolf 2 and wolf 4 exploration also increased when food was hidden around the enclosure. In accordance with previous studies [18,19,20,46,47], this finding suggests that the relationship between feeding enrichment and exploratory behaviour is more linear and less affected by individual variables than the other behavioural indicators we assessed.

Overall, both feeding enrichment programs seemed to be effective at positively modifying our wolves’ behaviour. However, inconsistency of behavioural responses among wolves indicates that individual variables might have qualitatively and quantitatively affected the beneficial effects of enrichment. Previous studies also found a high inter-individual variability in behavioural responses to feeding enrichment [18,48]. Since all of our experimental subjects were males with equal rearing conditions, this finding cannot be attributed to gender or rearing differences, as suggested by Cummings et al. [18] in their study on maned wolves. In our case, it is most likely explained by individual temperament differences and pack social dynamics [16,48,49,50].

A major limitation of this study is the small size of the sample, which prevents us from drawing any conclusion at the species level. Studies that investigate enrichment programs in zoo animals are often performed on small samples [18,34,51,52,53] and in specific environments that are likely not representative of the entire zoo population. Being opportunistic, our study presents these limitations. Our findings should be regarded as preliminary and further studies should be performed on larger samples and different captive environments.

In addition, each enrichment program was provided for 2 weeks only. The lack of differences between behaviours displayed in the first and second week of both enrichment phases suggest that, although the novelty of the enrichment decreased, it maintained beneficial effects in the analysed period. This is relevant for zoo animal caretakers, as their investment seems to have persistent benefits. However, 2 weeks may be a sufficient amount of time to determine short-term effects, but not enough to assess a possible long-term impact. This should be investigated in future studies by extending periods of enrichment.

Lastly, for practical reasons we could not assess any physiological indicator of acute or chronic stress, which could have helped us draw a clearer picture of the internal changes that each animal underwent during the study and their link with the behaviours observed [54,55,56].

### 4.2. Visitors

The number of visitors and the duration of permanence in front of the exhibit have often been used as indicators of their interest in zoo exhibits [22,23,35]. While the latter may be a more reliable parameter, the former may be more strongly affected by other variables such as season, day of the week, time of the day and weather [57]. This may explain why visitor number was higher in the final control phase than during the first enrichment phase. Nonetheless, our overall findings suggest that visitors were more interested in the exhibit during feeding sessions and during enrichment phases, especially when novel non-naturalistic objects were involved. In the latter case both number and time of permanence in front of the exhibit increased significantly. In previous studies, greater visitor interest in the exhibit has been linked to higher levels of animals activity [24,26,58]. However, although we found visitor number to be positively correlated with the number of active wolves at a given time, no significant difference in animal activity levels across experimental phases was detected. Other variables, such as the type of enrichment used [59] or the type of behaviour elicited [28,38,59] may also affect visitors’ interest in the exhibit.

Interestingly, those experimental conditions that increased visitors’ interest also enhanced their perception of captive wolf welfare. In fact, among daily observation sessions, the highest scores for animal welfare-related questions were obtained from those visitors who observed the wolves during feeding times, regardless of the use of enrichment. This result reflects those of previous studies in which visitors’ perception of captive wolf welfare improved by observing the animals perform active natural behaviours, such as feeding [28,29]. Furthermore, when we compared responses obtained from visitors who observed the wolves during feeding times, across all four experimental conditions, we found that higher scores for welfare-related questions were obtained when enrichment was provided, especially the non-naturalistic type. Previous studies [30,60,61] did not find any differences in the effects of naturalistic versus non-naturalistic enrichment on visitors’ perception of the animals’ well-being. However, some methodological differences, such as type of enrichment used [30,60,61], species involved [30,60,61], partial or total lack of complementary data on the animals’ behaviour [30,60] may explain the different results.

More importantly, visitors seemed to be aware of the implications that enrichment might have had on the wolves’ welfare. Price et al. [29] suggest that visitors’ perception of animal welfare may in turn be influenced by their own perception of zoos’ commitment at caring for their animals. In our study, artificial enrichment objects may have rendered the zoo’s commitment more evident in the eyes of the visitors.

Although this assumption should be further investigated with more specific questions in future studies, it may also explain why visitors who had higher perception of the wolves’ welfare also had a more positive perception of the educational role of zoos [59]. On one hand, negative emotions elicited by perceiving animals suffering may generate a deep sense of distrust in the zoo as an animal preservation institution, thus diminishing the value of its educational role [8]. On the other hand, animals stimulated to engage in natural behaviours, such as foraging and feeding, may not only be perceived as happier by visitors [8], but they may objectively provide more information about the species’ behavioural repertoire, if compared with inactive or stereotyping animals [29].

Findings from previous studies indicate that visitors who observe animals perform active natural behaviours show greater appreciation for the species’ biological significance and greater conservation intent [8,38,62]. According to that, our visitors showed a more positive attitude towards wolf conservation issues when they observed the animals during feeding times, regardless of the use of enrichment. However, feeding enrichment, whether naturalistic or non-naturalistic, failed to further improve visitors’ attitude towards wolf conservation. Due to the increasing wild wolf population, Italy is currently going through a period of intense political debate over wolf conservation measures. Actual or spurious episodes of attacks to livestock and even citizens reported by the media are likely to affect people’s attitude on the matter. This may explain why, in our study, visitors did not seem to change their perception of wolves as a threat to humans and human activities. Finally, it should be taken into account that the wolf is a historically controversial species. The cross-generational “evil wolf” myth embedded in the occidental culture may represent a significant obstacle for a mind shift on attitude towards wolf conservation [63]. Similar studies on other species or conducted in other countries may lead to different results.

## 5. Conclusions

Overall, both hiding food within the enclosure and providing animals with novel artificial feeding objects appeared effective at modifying the behaviour of our wolves in a way that suggests an increased level of welfare. However, inconsistency in results across experimental subjects indicates that individual variables play an important role in determining the degree and the type of behavioural response to feeding enrichment. Among the behavioural indicators assessed, an increase in exploration activity when novel feeding objects where provided was the only change in behaviour common to all the wolves.

Feeding enrichment, especially when artificial objects were used seemed to be effective at increasing visitors’ interest in the exhibit. Visitors’ perception of captive wolf welfare and the educational role of zoos, as well as their attitude towards wolf conservation issues were more positive when they observed the wolves during feeding times, regardless of the use of enrichment. Feeding enrichment, especially the non-naturalistic type, further improved visitors’ perception of captive wolf welfare. On the contrary, it failed to modify visitors’ attitude towards wolf conservation issues. In order to confirm this study’s findings future research on the effects feeding enrichment on wolves and visitors should be conducted on larger animal samples and in different countries.

## Figures and Tables

**Table 1:** Description of wolf behavioural categories grouped in macrocategories used for statistical purposes. Social interactions were categorized as either positive or negative. All behaviours, except stereotypies, were categorized as either active or inactive.

Behavioural Category	Description
Stereotypy	An apparently functionless fixed behavioural pattern that is repeatedly performed for at least three consecutive times
**Inactive**	
Sleeping	To lie down with closed eyes
Inactive	To sit or lie down with open eyes
**Active**	
Solo active	To stand motionless or move without interacting with other wolves
Solo play	To tug, chase, pull to pieces, jump on objects. To run alone, chase own tail, lie supine and roll or squirm in a playful manner
Exploratory	To sniff ground, objects, trees and plants, when not aimed at the acquisition of food
Foraging	To move food around, cache, dig and sniff, when aimed at the acquisition of food
Feeding	To take food into the mouth and swallow it
Drinking	
Marking	To urinate with raised leg
Self-directed	To nip, lick or scratch its own fur or skin, rub against a tree, stretch
**Negative social interactions**	
Dominant	To stand tall with rigid posture and tail, stand over another wolf with tail high, grab the muzzle of another wolf while maintaining a rigid posture
Submissive	To lie on the back with tail between legs, crouch
Aggressive	To show bare teeth, growl, snap, bite, attack, fight
**Positive social interactions**	
Affiliative	To sniff another wolf, lick another wolf, rub the muzzle against a wolf, rub the muzzle or body one another, greet, stand or lie close while wagging tails, put a paw or the head on another wolf while keeping tail down or wagging, stand over another wolf with tail down, chorus howl
Play	Two or more subjects that engage in motor patterns such as chase, run around one another, kick, jump and even jaw spar, snap or bite with not enough pressure to cause injury, play invitation

**Table 2:** Demographic information for questionnaire respondents.

Item/Response	Frequency	Percentage (%)
**Gender**		
Female	344	54.6
Male	280	44.4
**Age**		
<20	59	9.4
20–29	184	29.2
30–39	176	27.9
40–49	133	21.1
50–59	43	6.8
>60	32	5.1
**Ever been in a zoo before**		
No	47	7.5
Yes, more than once	476	75.6
Yes, once	106	16.8
**Nationality**		
Italian	440	69.8
British	30	4.8
American	20	3.2
French	13	2.1
Dutch	11	1.7
Romanian	11	1.7
Bulgarian	9	1.4
German	9	1.4
Russian	9	1.4
Irish	6	1.0

Nationalities below 1% were not reported.

**Table 3:** Likert-scale items and scoring system.

Items	Score (Level of Disagreement/Agreement)						
(1) I would love to spot a wolf in the wild	S. D.	1	2	3	4	5	S. A.
(2) Wolves are mean animals *	S. D.	1	2	3	4	5	S. A.
(3) Wolves are very dangerous to humans *	S. D.	1	2	3	4	5	S. A.
(4) Wolves in zoos behave like they do in documentaries	S. D.	1	2	3	4	5	S. A.
(5) Wolves in the wild are a serious threat to livestock *	S. D.	1	2	3	4	5	S. A.
(6) It is important to have wolves in zoos for education purposes	S. D.	1	2	3	4	5	S. A.
(7) Wolf reintroduction programs should be implemented in those areas from where the wolf disappeared	S. D.	1	2	3	4	5	S. A.
(8) Wild wolves that prey on cattle should be systematically eliminated *	S. D.	1	2	3	4	5	S. A.
(9) Illegal killing of wild wolves should be severely punished	S. D.	1	2	3	4	5	S. A.
(10) Wolves in zoos make me feel sad *	S. D.	1	2	3	4	5	S. A.
(11) The level of welfare of wolves in zoos is worrisome *	S. D.	1	2	3	4	5	S. A.

S.D. = strongly disagree, S.A. = strongly agree. Negative items are marked with an *.

**Table 4:** Component correlation matrix obtained for principal component analysis with promax rotation on questionnaire items.

Component	1	2	3
1	1.000	−0.158	−0.138
2	−0.158	1.000	0.257
3	−0.138	0.257	1.000

**Table 5:** Results of the principal component analysis and promax rotation carried out on items of the questionnaire. For each item, the load on each component has been reported.

Item	Component		
	Zoo Wolf Welfare	Wolf as a Threat	Wolf In-Situ Conservation
Wolves in zoos make me feel sad	**0.743**	−0.196	−0.114
It is important to have wolves in zoos for education purposes	**0.724**	−0.156	−0.037
The level of welfare of wolves in zoos is worrisome	**0.693**	0.012	0.042
Wolves in zoos behave like they do in documentaries	**0.578**	0.000	−0.239
Wolves are very dangerous to humans	−0.085	**0.814**	0.144
Wolves are mean animals	−0.120	**0.772**	0.163
Wolves in the wild are a serious threat to livestock	−0.065	**0.598**	0.242
Wolves reintroduction programs should be implemented in those areas where the wolf disappeared	0.044	0.063	**0.737**
Illegal killing of wild wolves should be severely punished	−0.088	0.175	**0.732**
Wild wolves that prey on cattle should be systematically eliminated	−0.209	0.307	0.557
I would love to spot a wolf in the wild	−0.195	0.459	0.512

Loadings with an absolute value greater than 0.5 (and lower than 0.25 for another component) are in bold.
